# Are Retail Outlets Complying with National Legislation to Protect Children from Exposure to Tobacco Displays at Point of Sale? Results from the First Compliance Study in the UK

**DOI:** 10.1371/journal.pone.0152178

**Published:** 2016-03-28

**Authors:** Douglas Eadie, Martine Stead, Anne Marie MacKintosh, Susan Murray, Catherine Best, Jamie Pearce, Catherine Tisch, Winfried van der Sluijs, Amanda Amos, Andy MacGregor, Sally Haw

**Affiliations:** 1 Institute for Social Marketing, School of Health Sciences, University of Stirling, Stirling, United Kingdom; 2 School of Health Sciences, University of Stirling, Stirling, United Kingdom; 3 Centre for Research on Environment Society and Health, School of GeoSciences, University of Edinburgh, Edinburgh, United Kingdom; 4 Child and Adolescent Health Research Unit, University of St Andrews, St Andrews, United Kingdom; 5 Centre for Population Health Sciences, Institute of Population Health Sciences and Informatics, University of Edinburgh, Edinburgh, United Kingdom; 6 ScotCen Social Research, Edinburgh, United Kingdom; University of Chieti, ITALY

## Abstract

**Background:**

From April 6^th^ 2015, all small shops in the UK were required to cover up tobacco products at point of sale (POS) to protect children from exposure. As part of a larger 5-year study to measure the impact of the legislation in Scotland, an audit was conducted to assess level and nature of compliance with the ban immediately following its introduction.

**Materials and Methods:**

A discreet observational audit was conducted 7–14 days post implementation which took measures of physical changes made to cover products, server/assistant practices, tobacco signage and advertising, and communication of price information. The audit was conducted in all small retail outlets (n = 83) selling tobacco in four communities in Scotland selected to represent different levels of urbanisation and social deprivation. Data were analysed descriptively.

**Results:**

Compliance with the legislation was high, with 98% of shops removing tobacco from permanent display and non-compliance was restricted almost entirely to minor contraventions. The refurbishment of shops with new or adapted tobacco storage units resulted in the removal of nearly all commercial brand messages and images from POS, dropping from 51% to 4%. The majority of shops stored their tobacco in public-facing storage units (81%). Most shops also displayed at least one generic tobacco message (88%).

**Conclusions:**

Compliance with Scottish prohibitions on display of tobacco products in small retail outlets was high immediately after the legislation implementation date. However, although tobacco branding is no longer visible in retail outlets, tobacco storage units with generic tobacco messages are still prominent. This points towards a need to monitor how the space vacated by tobacco products is utilised and to better understand how the continuing presence of tobacco storage units influences people’s awareness and understanding of tobacco and smoking. Countries with existing POS bans and who are considering such bans should pay particular attention to regulations regarding the use of generic signage and where within the retail setting tobacco stocks can be stored.

## Introduction

This paper presents results from the first study to examine compliance with UK legislation to protect children from exposure to tobacco displays at point of sale (POS). Displays of tobacco at POS are one of the few forms of marketing available to the tobacco industry in many countries and are particularly important for the industry in countries that have restrictions on tobacco advertising and sponsorship [1]. Research has shown that exposure to POS displays ‘normalises’ tobacco use [2,3] and increases susceptibility to smoking, experimentation, and initiation into smoking, particular among children and young people [4,5,6]. Studies of adults also suggest that POS advertising increase impulse cigarette purchases [7].

Consequently, Article 13 of the WHO Framework Convention on Tobacco Control, which came into force in 2005, advocates the complete ban on any display of tobacco products at points of sale [8]. The UK is one of the most recent jurisdictions to introduce a complete ban, through the Health Act 2009 [9] which covers England, Wales and Northern Ireland, and the Tobacco and Primary Medical Services (Scotland) Act 2010 [10] which covers Scotland. The legislation applies to all businesses selling tobacco products to the public and covers both the display of products and communication of price information.

**Fig 1:**
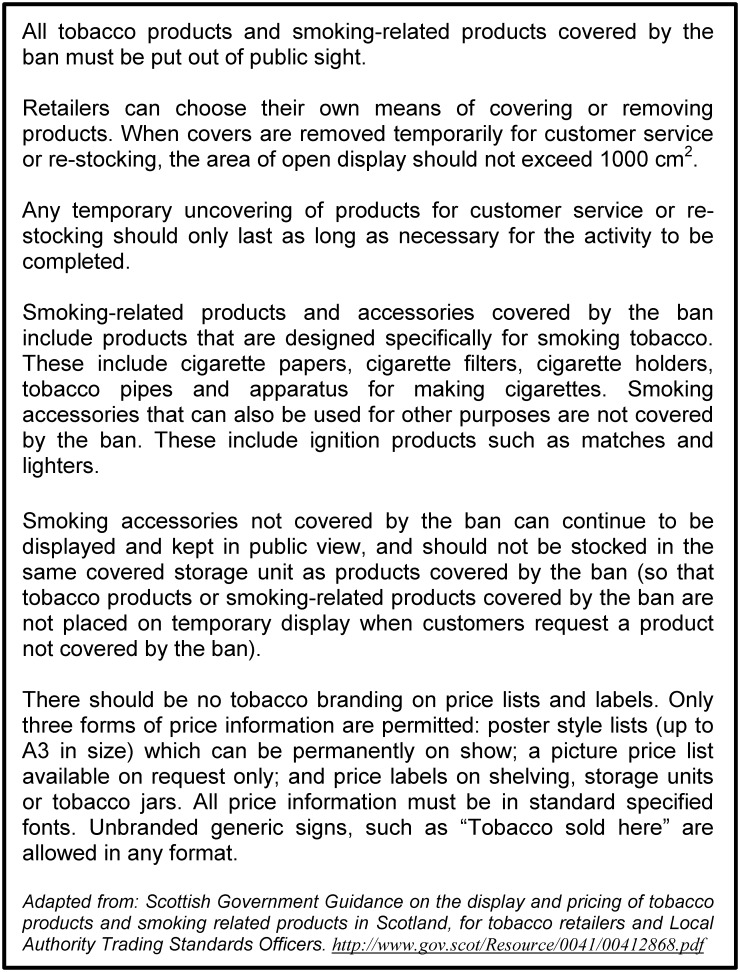
Summary of the implementation guidance for retailers in Scotland.

Since April 6^th^ 2015, all small retail outlets in the UK (premises with a sales floor area not exceeding 280 square metres) have been required to cover up tobacco products at POS. Supermarkets have been required to comply with the legislation since 2012 in England, Wales and Northern Ireland, and 2013 in Scotland. The legislation implemented in April 2015 applies to small grocery stores, confectioners/tobacconists/newsagents (CTNs), petrol station stores, off-licences (alcohol stores), and fast-food/takeaway outlets across the UK. These comprise the vast majority of retail sources of tobacco products, the only other legitimate source prior to the ban being mobile vans, a small proportion of which were registered to sell tobacco. Regulations concerning implementation of the legislation differ slightly in different parts of the UK (for example, the permitted maximum size of coverings is smaller in Scotland where the legislation also coveres smoking-related products). This study examined compliance by small retailers in Scotland. A summary of the implementation guidance supplied to retailers in Scotland is provided in Fig 1 [11].

It is important to assess compliance with new policies for a number of reasons. Poor compliance may reduce or undermine the effectiveness of the policy in bringing about its intended outcomes (in this case, the denormalisation of tobacco and reducing smoking prevalence, particularly among young people). Poor compliance may also indicate where additional interventions are needed in order to support adoption and implementation. This study is the first assessment of compliance in the UK in the period immediately after the implementation date.

## Materials and Methods

The data were collected as part of the DISPLAY study, a longitudinal study designed to evaluate the impacts of the legislation prohibiting POS tobacco advertising in Scotland [12]. For this paper, data were collected through an observational audit of retail outlets selling tobacco in four communities in Scotland 7–14 days after the display ban came into force in small shops. The aim was to assess level and nature of compliance with the ban immediately following its implementation. Whilst there were regulations governing the use of tobacco advertising at point of sale prior to the legislation, there were no prior restrictions on the way tobacco products could be displayed.

The observations comprised a discreet audit of all non-mobile retail outlets selling tobacco in the four study communities using a structured protocol. Each community was defined as the catchment area of one of four secondary schools. Candidate schools were initially identified from those in the central belt of Scotland with a school roll of over 1000 which were non-denominational, with an ethnic minority population of less than 10%. Approximately 43% of secondary pupils in Scotland attend large schools with school rolls over 1000. The schools were then selected based on levels of urbanisation and social deprivation. Each school’s deprivation level was estimated by calculating the mean (population-weighted) deprivation score (using the Scottish Index of Multiple Deprivation (SIMD) [13]) of the data zones (a data zone is the key small-area statistical geography in Scotland) that fell within the school catchment boundary. Two schools were recruited with high levels of socio-economic deprivation and two that had medium, low levels of deprivation. Mean SIMD scores for the four communities ranged from 11.6 to 32.3. To contextualize this, the mean raw SIMD score for all data zones in Scotland is 21.70 with a range of 0.94 to 89.89. Schools represented three of six classes of community as classified by the Scottish Government Urban/Rural Classification which combines measures of population and accessibility [14]. Two were selected from ‘large urban’ areas (class 1), one from ‘other urban’ areas (class 2) and one from ‘accessible small town’ areas (class 3). These data indicate that approximately 82% of pupils in Scotland attend schools located in these areas.

The total number of small retailers selling tobacco (n = 83) within each community ranged from 14 to 36, with the rate of small outlets per 10,000 population (10–19 years) varying between 49.6 and 81.0. Five categories of retail outlet were included: CTNs (confectioners, tobacconists and newsagents), grocery/convenience stores, petrol station/garage forecourt stores, off-licences (liquor stores) and fast food/take-away outlets. The sample was identified from the Scottish Tobacco Register (www.tobaccoregisterscotland.org), followed by field inspections of all streets in the communities to verify registered retailers and identify any unregistered retailers.

All of the observations were conducted by a single researcher (the lead author) making field visits in each study community. Data collection was facilitated by making a token purchase to allow close observation of the tobacco storage space, and by requesting the server/assistant on duty to advise on the cheapest tobacco brand currently stocked in order to assess the communication of price information. Audit protocols were completed away from the retail sites immediately after each observation. Where gaps or missing data emerged these were addressed by an immediate follow-up visit to the study outlet. The protocol was piloted in a small number of shops outside of the study communities by the same researcher responsible for conducting the audit. The researcher had extensive experience of observational research; no additional training was required.

Compliance with the ban was assessed using a combination of pre-coded and open-response fields to record observations for four categories of non-compliance: 1) permanent displays of products covered by the ban; recorded using a list of 7 pre-coded products, (e.g. cigarettes, loose tobacco, cigarette papers, cigarette filters); 2) effectiveness of storage unit maintenance and design at covering products; assessed using two pre-coded questions, ‘Does the cover mechanism limit the temporary display of products stored to an area of 1000sqcm?’ and ‘How effective is the storage unit design at concealing products from public view?’, incorporating an open-field to document how units were ineffective (e.g. open gaps between vertical slats); 3) exposure to display of banned products due to poor server practices; assessed by asking a dichotomous yes, no question, ‘Were any products covered by the ban left on temporary display for longer than was necessary to undertake a sale or legitimate action?’ and an open-response field recording observations for all yes responses; and 4) exposure to display of banned products due to poor storage practices; assessed using a pre-coded question ‘Did you note any products not covered by the display ban stored within the same covered storage space as products covered by the ban?’, supplemented by observations of banned products being stored in uncovered storage spaces. A summary of the main guidance given to shop keepers to support compliance is provided in Fig 1. A descriptive analysis was undertaken of frequency counts for prescribed items. New codes were developed for items identified from written observations.

Ethical approval for the study was provided by the Stirling University School of Management Research Ethics Committee. All data reported relate to observation of specific physical features of in-store retail environments accessible to the public. No identifiable information were collected relating to customers, shop keepers or their staff.

## Results

The study sample (n = 85) comprised: convenience stores (n = 54) CTNs (n = 15), garage forecourt shops (n = 10), off-licences (n = 3) and fast food take-aways/ fish and chip shops (n = 3). A total of 83 outlets were audited (two were closed at the time of visit) and all outlets were found still selling tobacco. No new outlets were identified in the four communities as selling tobacco.

Results for the three main aspects of compliance: physical changes made to comply with the ban, areas of non-compliance with covering displays, and provision of price information and tobacco signage are described below.

### 1. Physical Changes

Nearly all outlets (96.4%) had made some form of physical change to the way tobacco products were stored in order to comply with the ban (Table 1). Around four-fifths (80.7%) opted to place tobacco products in a new professionally designed storage unit or an adapted tobacco gantry. Most of these remained in a prominent position at eye level immediately behind the till-point. Seven shops (8.4%) had made ‘do-it-yourself’ adaptations to the existing display gantry, while the remaining six shops (7.4%) had removed their tobacco stock completely away from public view, typically placing their tobacco products in pull-out drawers under the service counter. Three of the six shops that had moved products from public view still retained original purpose designed tobacco gantries with their generic tobacco signage on display. The Scottish legislation does not regulate the use of generic signs referring to tobacco.

**Table 1:** Physical changes made to comply with the ban (n = 83).

	%	N
Outlets not making physical changes	3.6	3
Outlets making physical changes	96.4	80
Type of change made:		
*Professionally adapted or designed storage unit*	*80*.*7*	*67*
*DIY adaptation to existing storage unit*	*8*.*4*	*7*
*No identifiable storage unit / Products completely removed from public view*	*7*.*2*	*6*

The audit also assessed the types of cover mechanism used on storage units to conceal tobacco products from view. Nearly three-quarters of shops (71.1%) used horizontal hinged gravity flaps of the type used in large supermarkets in the UK; 8.4% used vertically suspended blinds which enabled retailers to access products from between the individual slats; and 9.6% used a combination of these two devices, typically relying on flaps to conceal tobacco products and slats to conceal smoking accessories covered by the Scottish legislation such as cigarette papers.

### 2. Areas of Non-compliance with Covering Displays

Compliance with the legislation was good, with most contraventions only minor in nature. Instances of non-compliance fell into one of four categories: permanent displays of products; storage unit maintenance and design; storage practices; and server/assistant practices (Table 2).

**Table 2:** Areas of non-compliance with covering displays (n = 83).

	%	N
Permanent displays of products:		
*Tobacco products continued to be displayed*	2.4	2
Smoking-related products covered by the ban continued to be displayed*	15.7	13
Storage unit maintenance and design:		
*Vertical slats too narrow/short to cover products*	8.4	7
*Products on partial view due to poorly fitted horizontal flaps/flaps in a poor state of repair*	6.0	5
*Individual covers exceeding the permissible area*	3.6	3
Storage practices:		
*Products not subject to the ban viewed in the same covered storage space as products subject to the ban*	20.5	17
*Storage cartons visible*	8.4	7
Server/assistant practices:		
*Multiple flaps opened simultaneously*	6.0	5
*Flaps /slats left open*	2.4	2
*Other*	1.2	1

*Smoking-related products and accessories covered by the ban include products that are designed specifically for smoking tobacco. These include cigarette papers, cigarette filters and tobacco pipes. Smoking accessories that can also be used for other purposes are not covered by the ban. These include ignition products such as matches and lighters.

#### Removal of permanent displays

Two outlets (2.4%), a small independent café and grocers store and a local fish and chip shop / fast-food outlet, continued to display their tobacco products in direct contravention to the ban. Both stocked relatively small ranges (3–4 cigarettes brands) which were displayed on generic shelf units alongside other non-tobacco products. Thirteen outlets (15.7%) had permanent displays of other smoking-related products, typically cigarette papers and filters. These were normally displayed on open shelves often below eye-level and were relatively unobtrusive and difficult to spot.

#### Storage unit maintenance and design

Seven outlets (8.4%) had vertical slats that were only partially effective at concealing products: either leaving gaps between the slats to reveal the products behind, or gaps at the base of storage units with products on view as the slats were too short. Five outlets (6.0%) revealed banned products due to flaps that were poorly fitting or in a poor state of repair. Two of these outlets had one or two flaps missing with the products in the storage space behind on permanent view. Three outlets (3.6%) used flaps that appeared to reveal an area greater than was permitted under the ban (no more than 1000cm^2^ at any one time). One of these outlets relied upon the original tobacco gantry’s aluminium security shutter to conceal tobacco products from public view; the roller-shutter was lifted by the server/assistant to its full height to view the full range of products at the time of visit.

#### Storage practices

The display legislation requires that smoking-related products and accessories covered by the ban, such as pipes and paraphernalia for making cigarettes, are not stored within the same covered space as other products and smoking accessories that can also be used for other purposes for example. A total of17 outlets (20.5%), were found to breach this part of the legislation. In most cases these were shops where matches and lighters were stored alongside cigarette papers and cigarette filters. In seven other cases (8.4%) retailers were observed storing cartons containing tobacco products on an open storage unit or shelf underneath the main storage unit. In some cases cartons were partially open with branded packs on view, or individual packs were left out on the back counter awaiting storage.

#### Server/assistant practices

Eight minor contraventions were observed. In five cases (6.0%) a server/assistant was observed opening more than one flap at a time in order to maximise the range of products on view and to assist the customer make a product choice. In two other outlets (2.4%), storage unit flaps and blinds had been left open at the time of visit, and in one other case a retailer’s young daughter was observed behind a set of vertical slats playing with the tobacco stock.

### 3. Price Information and Tobacco Signage

Compliance with the legislation governing the communication of price information was high with few contraventions recorded; one tobacco price list appeared to exceed the restrictions on font size and page length, while another outlet had hand-written product labels on storage flaps that clearly exceeded the permitted font size.

The audit also examined how prices were communicated. Twenty six outlets (31.3%) had product labels with prices on storage units, most of which were not legible from the customer side of the counter (Table 3). Sixteen outlets (19.3%) reported having a price list for tobacco products, although six of these could not find their list to view at time of visit. Fifty outlets (60.2%) had no price information on display for customers; some outlets had price stickers on their shelf strips but these did not include brand information, and some had brand labels on the front on storage flaps but these did not include price information. With the exception of price lists, most of the product and price information that was visible appeared to be intended for shop staff use.

**Table 3:** Provision of price information and tobacco signage (n = 83).

	%	N
Availability and communication of price information:		
*No written pricing information available*	60.2	50
*Price labels on storage units*	31.3	26
*Price lists on view / available on request*	19.3	16
*Reluctance to provide price information*	8.4	7
In-store industry and generic tobacco messages:		
*Generic tobacco signage*	88.0	73
*Manufacturers’ insignia*	49.4	41
*Brand-specific tobacco advertising*	3.6	3

The majority of outlets communicated price information verbally to customers, often opening individual flaps to confirm this information; most outlets continued to rely on price-marked products and used the price flashes on the side of packs to confirm prices. All of these practices were compliant with the legislation, with the exception of some cases of servers/assistants opening more than one flap at a time. In seven cases (8.4%) the server/assistant expressed a reluctance to reveal or communicate any price information when asked about the ‘cheapest’ cigarettes stocked, either suggesting that this was not permitted under the ban or that s/he could only communicate prices if asked about a specific brand. This response appears to stem from retailers’ interpretation of the guidance prohibiting retailers from promoting specific brands.

The audit also monitored the use of tobacco signage following implementation. The majority of outlets (88.0%) displayed at least one generic tobacco message on the storage unit top panel or flap fronts (these are permitted under the legislation). Typical messages included ‘Tobacco on sale here’, ‘Rolling tobacco’, ‘Cigarettes’, ‘Cigars’ ‘Tobacco’.

Following the introduction of the legislation nearly half of all shops (49.4%) were recorded as now carrying the corporate insignia of specific tobacco manufacturers in the form of colour scheme or company labels on professionally designed storage units. More specifically, it was noted that shops with storage units supplied by Imperial Tobacco had units in the same shade of red as that featured in the Imperial Tobacco website and on the trade literature produced by Imperial Tobacco for retailers. Similarly, storage units supplied by the Gallagher Group carried the corporate insignia of its parent company JTI (Japanese Tobacco International), although the small size of these insignia means it is likely only to be legible to staff working from behind the service counter. The Scottish display ban does not include any provisions for the use of corporate advertising. In addition to corporate insignia, three outlets (3.6%) were recorded as retaining brand-specific tobacco advertising on their storage units; all three had shelf strip advertising and two carried A4 posters on their top panels for specific cigarette brands and related products. All three were compliant with the existing legislation governing tobacco advertising at POS in Scotland which does not include advertising for smoking-related products and accessories. The same legislation does not include provisions for corporate advertising.

## Discussion

This study in four communities of small retail outlets’ response to the legislation in Scotland banning POS tobacco displays found that compliance with the new regulations was high in the period immediately after the legislation implementation date. While the legislation in Scotland differs slightly from that in other parts of UK—for example, only the Scottish legislation covers some smoking-related products—it is believed that results relating to tobacco products can be generalised for the whole of the UK. The high levels of compliance achieved by small shops in the Scottish study would also appear to be broadly consistent with that achieved by larger stores in the four study communities (unpublished), although the small numbers involved (n = 10) precludes any detailed analysis or comparisons.

Compliance studies in other countries which have implemented complete bans or restrictions on the visibility of tobacco products and advertising at POS have found similarly high levels of compliance. For example, an evaluation of legislation in Victoria, Australia, found almost universal compliance, with 94.1% of stores observing the full ban three to four months after the enactment of the legislation [15]. Similar, bans in Norway and Ireland recorded compliance at rates as high as 97% post implementation [16,17]. Historically, where partial restrictions have been introduced, compliance has been lower, suggesting that complete bans are likely to be both more effective and easier to implement [18]. The high level of compliance found in this study demonstrates that it is possible to implement comprehensive bans across a wide range of retail outlets.

The majority of retailers sought to comply with the ban by installing purpose-designed covers and fittings, many of which were provided by tobacco companies. Interestingly, relatively few retailers (8.4%) were recorded as having used ‘do-it-yourself’ adaptations to conceal products. Those that did use ‘do-it-yourself’ adaptions were likely to be less compliant with the legislation.

Many of the storage unit fittings supplied by the industry carried the corporate insignia or colour scheme of a specific tobacco manufacturer. In their current form and current context these corporate messages appear to be designed with the retail trade rather than customers in mind. It is suggested that close monitoring of how the covers on storage units supplied to retailers are used is required in order to assess how corporate messaging evolves and whether it establishes any relevance for customers and young people.

As well as promoting compliance through the installation of effective covers, purpose-designed fittings have also been responsible for the removal of nearly all point of sale advertising for tobacco brands and smoking-related accessories. Only a small number of advertisements were recorded after the ban came into force, all of which were found in stores where retailers had either largely ignored the ban or had relied upon a ‘do-it-yourself’ adaption in order to comply. Prior to the legislation, just over a half (51%) of all outlets featured brand advertising for tobacco and smoking-related products on tobacco display gantries (unpublished). Existing UK legislation limits the amount of tobacco advertising allowed at POS to a single A5 poster or its equivalent [19]. The advertising of smoking accessories such as cigarette papers are exempt from this legislation. Cohen et al [20] found a similar effect with the introduction of a display ban in Ontario, Canada which resulted in a decrease in tobacco promotions.

Although compliance with the legislation was found to be high, tobacco continues to maintain a strong visible presence in small shops, with most retailers opting to retain their existing tobacco unit rather than moving tobacco products to other parts of the store completely away from public view. These new and adapted storage units were still clearly visible, with most located in a prominent position behind the service counter and most continuing to carry generic signage promoting tobacco availability. These features continue to convey the message that tobacco is an important retail product.

While nearly all outlets attempted to comply with the ban, two outlets displayed their tobacco products in direct contravention to the ban, a small independent café and grocers store and a local fish and chip shop / fast-food outlet. Neither had previously held a large stock or had a display unit, which suggested that tobacco sales were relatively unimportant to their business. The retailers in question may have felt the legislation did not apply to them or that they were less likely to be caught or penalised. In cases such as these where contraventions are blatant, active enforcement is required. These findings also underline the need for monitoring all types of outlet, not just those that rely heavily on tobacco sales.

Most other cases of non-compliance were relatively minor and temporary in nature and it is suggested that lack of awareness is likely to explain many of these breaches, most of which could be remedied by careful monitoring and provision of advice and training to retailers and staff.

There are some limitations of the study methodology. The data collected are not nationally representative although they were obtained in four communities with differing levels of social deprivation, and degrees of urbanisation. The relatively small sample size (n = 83) meant that it was not suited to analysis by area and shop type and to more complex analysis such as random-effect logistic regression [21]. This was experimented with and results confirmed that the four clusters were not large enough for meaningful analysis. In addition, the data collection methods used can be subject to observer recall error. However, the use of memory aids and completion of protocols immediately after observation minimised this type of error, as did the ability to make repeat visits if required to verify and collect any missing data. Other methods involving photography and video were piloted, but proved unreliable and not necessarily capable of capturing all the information required for the audit. Assessment of some breaches, for example whether or not storage unit covers complied with the size requirements of 1000 square centimetres, was reliant on observer judgement rather than detailed measurement.

In summary, compliance with new legislation prohibiting the public display of tobacco products in retail outlets was high in the period immediately after the legislation implementation date. Whilst it is not possible to determine the reasons for high compliance, factors which would appear to be important included: the decision to introduce a complete ban which helped to reduce any ambiguity regarding compliance; giving small independent shop keepers who were less well-resourced than large supermarket chains an extra 24 months to acclimatise and prepare; and requiring all shop keepers to register their intention to sell tobacco on a national database during the lead-up to the legislation. A physical check of all fixed retail outlets in the four study communities carried out prior to the shop audits identified only one tobacco retailer who had failed to register on the database. In public health terms, the high rate of compliance is encouraging. However, although tobacco branding is no longer visible in retail outlets, the decision by most retailers to continue to stock tobacco in large, prominently positioned storage units featuring generic tobacco messages means that tobacco still has a strong and ubiquitous presence in the retail setting raising questions about the messages this gives to customers and young people. Countries considering POS bans and countries where such legislation already exist should pay particular attention to regulations regarding where within the retail setting tobacco stocks can be stored, what size of storage unit should be permitted and how tobacco can be marketed to inform customers of its availability.

## Supporting Information

S1 File Minimal data set. (XLSX)
